# An elderly man presenting with an acute upper gastrointestinal bleed: a case report

**DOI:** 10.4076/1757-1626-2-7951

**Published:** 2009-08-12

**Authors:** Riaz A Agha

**Affiliations:** Department of Gastroenterology, Hinchingbrooke Health Care NHS Trust, Hinchingbrooke HospitalHinchingbrooke Park, Huntingdon, Cambridgeshire, PE29 6NTUK

## Abstract

An 80 year old man presented to the Accident and Emergency Department complaining of “black stools”, increasing shortness of breath, chest tightness and epigastric pain. An upper gastro-intestinal bleed was diagnosed and the patient was managed conservatively with aggressive resuscitation and close monitoring. An oesophogastroduodenoscopy found no cause for the bleeding which ceased and the patient was discharged with a general practitioner follow-up.

## Introduction

Acute upper gastrointestinal haemorrhage (AUGH) is a common medical emergency with a significant associated mortality [1] despite ongoing advances in its management [2].

Following decades of research, the introduction of risk stratification and prognostic scoring in the Rockall Score has allowed for the early division of this pool of patients into low or high risk of re-bleeding or death. This allows for the more effective management of resources and has brought greater awareness to the need for close monitoring of high risk patients who can decompensate suddenly on busy wards and admissions units.

## Case presentation

A retired 80 year old Caucasian man presented to the Accident and Emergency Department complaining of “black stools”, increasing shortness of breath, chest tightness and epigastric pain. Three days previously he had celebrated his birthday when he noted epigastric pain. This was similar in nature to the pain from his duodenal ulcers 20 years ago (managed conservatively at the time). The pain was relieved by eating and settled with simple analgesia and rest. From that point, the patient had black stools daily with decreasing exercise tolerance and shortness of breath on exertion. His past medical history included atrial fibrillation (AF), hypertension, and ischaemic heart disease with a bare metal stent inserted 6 weeks prior to admission in the left anterior descending branch. The patient was taking aspirin and warfarin for AF. The patient was a non-smoker, with minimal alcohol intake, a body mass index (BMI) of 25 and with no family history of note.

The patient was found to be normotensive at 130/80, but with a postural diastolic drop of 15 mmHg with a pulse of 80, decreased skin turgor, oxygen saturation of 98% on air and a respiratory rate of 16. Routine blood tests found that Haemoglobin (Hb) was 12.5 g/dL, with a urea/creatinine ratio >100 and an International Normalised Ratio (INR) of 2.3. On examination the patient was found to have mild tenderness in the epigastric region. The patient’s initial Rockall score was thus four which carries a 24.6% risk of mortality [3].

The patient was treated with normal saline IV fluids and his anticoagulants were stopped. Reversal of warfarin with vitamin K was not deemed necessary. Fluid and stool charts together with regular observations were initiated. Oesophogastroduodenoscopy (OGD) was scheduled to take place within 72 hours of admission. During the period prior to OGD, the patient’s blood pressure dropped to 110/70, pulse increased to 95 and Hb decreased to 10.3 g/dL. The patient did not become symptomatic and a blood transfusion was not performed although a group had already been taken. Fluid resuscitation and close observation continued during this period with good urine output and just one further episode of malaena. OGD was performed without complication and found no ulcers or bleeding points down to the fourth part of the duodenum. The patient’s Hb recovered to 13.5 g/dL and he became normotensive and euvolaemic. Anticoagulants were restarted and the patient was discharged four days after admission without further event with a GP follow-up scheduled.

## Conclusions

This case report illustrates the need for prognostic scoring, close observation and aggressive fluid resuscitation in the management of GI bleeds.

## Abbreviations

AF: atrial fibrillation
AUGH: acute upper gastrointestinal haemorrhage
BMI: body mass index
Hb: haemoglobin
INR: international normalised ratio
IV: intravenous
